# Combating the menace of antimicrobial resistance in Africa: a review on stewardship, surveillance and diagnostic strategies

**DOI:** 10.1186/s12575-022-00182-y

**Published:** 2022-11-23

**Authors:** Bashar Haruna Gulumbe, Usman Abubakar Haruna, Joseph Almazan, Ibrahim Haruna Ibrahim, Abdullahi Adamu Faggo, Abbas Yusuf Bazata

**Affiliations:** 1 Department of Microbiology, Federal University Birnin Kebbi, Kalgo, Kebbi State Nigeria; 2 Department of Medicine, Nazarbayev University School Medicine, Nursultan, Kazakhstan; 3 Faculty of Pharmaceutical Sciences, Ahmadu Bello University, Zaria, Nigeria; 4 Research Center for Cancer Biology, Graduate Institute of Biomedical Sciences, College of Medicine, China Medical University, Taichung City, 406040 Taiwan; 5 Department of Microbiology, Bauchi State University, Gadau, Bauchi State, Nigeria

**Keywords:** AMR detection, AMR surveillance, Antibiotic susceptibility testing, Stewardship, Diagnostics, Africa

## Abstract

The emergence of antibiotic-resistant pathogens has threatened not only our ability to deal with common infectious diseases but also the management of life-threatening complications. Antimicrobial resistance (AMR) remains a significant threat in both industrialized and developing countries alike. In Africa, though, poor clinical care, indiscriminate antibiotic use, lack of robust AMR surveillance programs, lack of proper regulations and the burden of communicable diseases are factors aggravating the problem of AMR. In order to effectively address the challenge of AMR, antimicrobial stewardship programs, solid AMR surveillance systems to monitor the trend of resistance, as well as robust, affordable and rapid diagnostic tools which generate data that informs decision-making, have been demonstrated to be effective. However, we have identified a significant knowledge gap in the area of the application of fast and affordable diagnostic tools, surveillance, and stewardship programs in Africa. Therefore, we set out to provide up-to-date information in these areas. We discussed available hospital-based stewardship initiatives in addition to the role of governmental and non-governmental organizations. Finally, we have reviewed the application of various phenotypic and molecular AMR detection tools in both research and routine laboratory settings in Africa, deployment challenges and the efficiency of these methods.

## Introduction

AMR is increasingly recognized as a major public health threat which has been estimated to cause at least seven hundred thousand deaths per year globally. In terms of economic impact, AMR has been projected to cost the global economy about one hundred trillion dollars per annum in addition to causing the loss of millions of lives if efficient measures are not put in place to tackle it [1, 2]. Arguably, AMR is a challenge that affects every continent and country regardless of their levels of development. However, in Africa, the problem of AMR is aggravated by a number of factors, including indiscriminate use of antibiotics, poor sanitary conditions, poor water quality, suboptimal health care system exasperated by crude diagnostic practices and lack of capacity-building programs, lack of access to quality antibiotics as well as poor surveillance and stewardship programs [3]. Further, in many parts of Africa, healthcare facilities are grossly inadequate, and where they exist, they are often antiquated, with insufficient diagnostic capability and anirreliable supply of reagents, leaving people with limited access to professional healthcare. In these situations, sick patients are left with options of self-medication, traditional treatment options, or seeking care from drugstore employees, some of whom have no medical training of any sort [4]. While some of these problems are linked to resource limitation and poverty, others simply demonstrate a lack of commitment on the side of governments.

However, recognizing the significance of AMR and its impact, the global health community has taken steps to contain the spread of AMR globally through improved disease prevention and control, as well as the promotion of rational antibiotic use. For example, the European Strategic Action Plan on Antibiotic Resistance was established in 2011 to create and implement policies to assist European Commission member countries in addressing the numerous causes of AMR, including coming up with twelve recommendations which have been adopted by other countries beyond Europe [5]. Similarly, to improve cooperation between Europe and the United States in the fight against AMR, the Transatlantic Taskforce on Antimicrobial Resistance (TATFAR) was founded more than a decade ago [6]. In the same vein, the United States Centre for Disease Control (US CDC) has established a national laboratory network to assist hospitals in quickly identifying drug-resistant illnesses and halting their spread. Unfortunately, these kinds of strategies are scarce in the African setting and where available, they are poorly implemented [2, 4]. For example, to date, out of the 36 African countries that have finalized their national action plans (NAPs) on AMR [7], 27 have received approval from the governments [8], while only Kenya, Burkina Faso, Nigeria, Côte d'Ivoire, Mozambique and South Africa were reported to have national AMR action plan currently implemented and actively monitored [9].

In the areas of surveillance systems, stewardship programs and the discovery of novel diagnostic tools, no significant progress has been made [10]. Based on the recent report from the World Health Organization (WHO) on AMR surveillance [7], 23 out of the 47 African member states enrolled in the Global Antimicrobial Resistance and Use Surveillance System (GLASS), and just 15 have reported data on the national surveillance program. A very low level of implementation of AMR guidelines has been reported in most African countries despite a surging AMR crisis on the continent. In a study, Craig and colleagues [11] have reported a lack of antimicrobial treatment guidelines that meet internationally established protocols across all African Union (AU) member states. This particularly sends a very bad signal in the aspect of AMR containment since the guidelines provide a chance to lessen the wrongful and excessive use of antibiotics in human health. The guidelines are crucial tools for ensuring the proper and optimal use of antibiotics, which can help prevent the development of antibiotic resistance. They include specific guidelines for treating infections and support clinical judgment. They have been found to decrease the incorrect administration of antibiotics and enhance the standard of care [9].

Similarly, the lack of sufficiently trained infection control personnel and the scarcity of robust and affordable point-of-care diagnostic facilities are some of the major factors identified as contributing to the problem of poor surveillance program implementation [10, 12]. To tackle the AMR problem and minimize its impact, the rational use of antibiotics in compliance with the established clinical guidelines that describe the rudiments of antibiotic use is advocated by expert organizations [11]. These kinds of advocacy can indeed be implemented at both the facility and community levels through stewardship programs. Unfortunately, in settings with limited resources struggling with a lack of trained professionals aggravated by the absence of guidelines, stewardship programs are unlikely to succeed. AMR awareness and advocacy strategies are important since they help to ensure that politicians, policy-makers, professionals, civil society actors, and the society at large understand why it is critically important to address AMR, including the vital role that AMR surveillance plays in these efforts.

In this review paper, we set out to provide a comprehensive assessment of the application of diagnostic tools, surveillance and stewardship programs in Africa with a view to understanding the progress made so far in the region as well as deciphering the level of compliance to the standardized AMR guidelines.

## AMR stewardship programs and initiatives in the African setting

The advocacy for antibiotics to only be given to patients who genuinely need them is arguably a crucial strategy in the fight against AMR [13]. To achieve this, the antimicrobial stewardship (AMS) program has been developed and implemented by many countries around the world. According to the WHO (2019) [14], AMS can be defined as “an organizational or system-wide health-care strategy to promote appropriate use of antimicrobials through the implementation of evidence-based interventions with the aim of optimizing the use of antibiotics and changing the prescription practice [14].” AMS program is one of the plans of the African governments in combatting the development of antibiotic resistance. Programs such as the AMS are relevant, particularly since studies have shown that in the African setting, there is a high fraction of antibiotics inappropriately used [15, 16]. Although there is a scarcity of data on the implementation of AMS programs in Africa, most of the AMS interventions implemented were only observable in a few African countries [17].

In the global survey of WHO in 2018, Mozambique, Nigeria, Ghana, Kenya, Tanzania, Zimbabwe and South Africa were the only African countries to have an action plan that aligns with the objectives and arrangements in the global action plan, with only Tanzania, Kenya and South Africa having AMS activities [18]. However, in the last two years, some progress has been made as the global survey of WHO has shown that among the African countries, only the Central African Republic reported no national AMR action plan, while Kenya, Burkina Faso, Nigeria, Côte d'Ivoire, Mozambique and South Africa were reported to have national AMR action plan currently implemented and actively monitored [9]. The WHO survey indicates that almost all the countries in Africa have already made their action plan nationally to mitigate the problem of AMR. For example, in 2017, Kenya created a plan of action toward the prevention and control of AMR with the strategic objectives of improving the awareness ofAMR, enhancing knowledge through research surveillance, infection prevention, improving antibiotics efficiency and infection reduction through sanitation [19]. In addition, they created the National Antimicrobial Stewardship Inter-Agency Committee (NASIC) and other technical committees with specific roles and responsibilities that will make up their national government coordination mechanisms.

At the facility level, there were AMS programs implanted in a few hospitals on the continent. For instance, in Southern Tanzania, the “Mbeya Antimicrobial Stewardship Team (MAST)” was developed through collaboration between a local hospital and South Carolina University with the aim of strengthening the order of antibiotics. Through the program, the widespread assumptions of frequent deviations from national recommendations for empiric therapy were confirmed by the structured examination of antibiotic ordering and administration. The team identified challenges associated with the antibiotic supply chain, such as problems with transportation, lack of finance, and frequently the government-owned supplier was unable to adapt to shifting pharmaceutical demand as some of the obstacles to optimum antibiotics use. The data from the program helped in providing informed recommendations and supported initiatives to engage hospital leadership and other stakeholders [19]. Furthermore, the team yielded positive results in providing and exchanging data on the prescribing habits in the region, accessibility of antimicrobials and the illustration of problems encountered by countries while upholding the guidelines [20]. In a similar scenario, George Hospital located in the Western Cape of South Africa, developed an AMR awareness program in 2015. The Western Cape Department of Health and the Improving Global Health (IGH), through the Leadership Development Program in the UK, assigned George hospital three healthcare professionals who would supervise the improvement of the antibiotic stewardship program in terms of awareness of AMS and staff participation with a view to achieving rational antibiotics use and better patient outcomes. The professionals have been rotating for 6-month periods to ensure that the quality of the program improved. It brought about increased awareness of AMS principles and an increase in staff participation during rounds which is critical in the implementation of AMS programs [21]. Similarly, a study conducted in a South African hospital revealed that the AMS program among pediatric patients increases the percentage of children managed based on the standard guidelines [22]. In another study, it was reported that the antimicrobial intervention of pharmacists was able to improve the timely administration of antibiotics across South African hospitals. In the study of Messina et al. [23] on pre-intervention and post-intervention among 33 hospitals in South Africa, patients were assessed based on the hang-time compliance from the time the written antibiotic order was made to its actual medication administration. The results of their study demonstrated a change in the hang time compliance from pre-intervention and post-intervention, thereby increasing the timeliness of antibiotic administration. Thus, pharmacists were considered to contribute to enhancing the AMS initiatives within the local hospital system in a resource-limited country.

Despite the initiatives in South Africa, AMS is yet to be fully implemented in primary healthcare facilities. There is a lack of continuing education among prescribers at the primary healthcare facilities and there were nearly non-existing healthcare advocacies and campaigns [24]. In Sub-Saharan African countries, AMS was the least prioritized [25], with only 32% of the countries reporting a national guideline towards proper antibiotic administration, while other countries reported uninhibited use of counterfeit and unregulated antibiotics [25]. Clearly, African countries still need to strengthen their implementation of the AMS programs through collaboration with all the stakeholders, including civil society organizations.

## The role of civil society organizations

Civil Society Organization (CSO) is “any non-profit, voluntary group which is organized on a local, national or international level to perform a variety of services and humanitarian functions. These include bringing citizens’ concerns to governments, monitoring policies, and encouraging political participation at the community level” [26]. CSOs being community-centered groups, have capacities to provide information at a community level which is a great help to the government in realizing the real AMR situation on the ground. Similarly, they disseminate information to the public in a more simplified manner which can be effective in spreading awareness. They also play a vital role in accelerating the awareness of AMR in African countries. CSOs have been reportedly involved in the battle against AMR by advocating AMR control. A study has shown that 35 CSOs in 37 countries across Africa are actively focusing on the four areas of “Africa Centers for Disease and Control Framework,” such as enhancing surveillance, delaying emergence, limiting transmission and mitigating harms from the infection of AMR. Half of the CSOs were found to be engaged in the creation and implementation of the NAP, although only three of them revealed that their work was based on national strategies [27]. In order to reduce antibiotic resistance even more, in collaboration with other CSOs, Action on Antibiotic Resistance (ReAct) uses a one-health approach while working with several stakeholders at different levels to support the development and execution of NAPs and work to increase public and professional knowledge of issues related to health, veterinary medicine, agriculture, and the environment across Africa [27]. In a recent review, Fracer et al. [27] have extensively looked at the roles of CSOs in reducing AMR transmission, lessening its harm in Africa. To date, despite the commendable involvement of CSOs in the fight against AMR, there is a need for more participation, particularly in the area of AMS. The African CSOs have potential to be involved in AMS-related activities due to their vital role. Thus, empowering these organizations could contribute meaningfully to solving the problem of AMR.

## The role of African governments and agencies on AMS and the fight against AMR

Several African countries have NAPs on AMR, signifying that the African governments have made efforts to tackle the issue and brainstorm ways to address them properly [28]. Reducing the prevalence of infection through efficient sanitation, hygiene, and infection prevention methods is one of the strategic goals of NAPs, as these are crucial to the fight against the spread of resistant microorganisms. A recent study revealed that 11 out of 15 African countries made their NAP available to the public each has provided strategic objectives and practical ways to achieve those [29]. In a similar scenario, Zambia also established a multi-sectoral NAP on AMR, which was aligned with the objectives of the implementation of the global action plan. The main aim is to address the spread of AMR in the Zambian context. Senegal, which is part of Sub-Saharan countries, also made efforts in antimicrobial rational use promotion by requiring a doctor’s prescription before using the antibiotics and assigned the Directorate of Pharmacy and Medicines to carry out activities such as raising awareness of the danger brought by taking over-the-counter drugs [30]. Similarly, Ghana tackled the issue of AMR through the establishment of the National Platform on Antimicrobial Resistance (NPAR). Prior to its establishment, various brainstorming sessions were conducted to identify ways to address the issue at a national level and the main aim of the organization was to partner with the government and civil society in order to develop and impose policies regarding the containment of antibiotic resistance.

Like in the area of AMS, the role of governments in coming up with policies to tackle AMR is evident in South Africa compared to other African countries. The journey of South Africa on AMR started in 2011 when a situation analysis revealed that the country faced a high burden of infectious diseases. In 2014, a national AMR strategy framework was developed by the Ministry of Health outlining the plan for combating AMR [31]. In the same country, an “AMR national strategy framework,” a one-health approach that focused on slowing the expansion and spread of AMR and improving antibiotic use appropriately for individual health, was created in 2018. In this framework, a committee saddled with the responsibility of coordinating intersectoral efforts, strengthening the AMR national surveillance system, ensuring that the laboratories provide consistent diagnostic standards, enhancing the country’s prevention and control as well as promoting the appropriate use of antimicrobials [32].

Aside from action plans, conferences and seminars were also conducted by the AU heads of states and governments in order to develop policies and programs to mitigate the impact of AMR on the continent. The AU also encourages each member state to provide funds for the training and retraining of the personnel to be assigned to handle AMR issues. The African Common Position on AMR encourages the member states of the AU on the appropriate use of antimicrobials, promoting and minimizing the sale of sub-standard and fake antimicrobials in each member state [33]. Moreover, they also encourage each healthcare facility to implement AMR control and prevention programs by adhering to international standards in hygiene and sanitation [33].

Indeed, various efforts have been made not only by the African governments as a whole but also by other stakeholder organizations to ensure the safety of the health of the African people. For instance, The Mapping Antimicrobial Resistance and Antimicrobial Use Partnership (MAAP) project led by the African Society for Laboratory Medicine (ASLM) in partnership with various African organizations also planned to ensure that they are able to provide data on controlling AMR and develop policies at the institutional, regional and national levels based on the data collected from the laboratories which have conducted clinical bacteriology testing [34]. Similarly, the administrative staff of the Ministry of Health of countries in West Africa worked together to conduct various seminars and workshops to tackle various issues on AMR. For instance, their 2017 seminar focused on discussing leading the future programs of African countries on AMR [30]. Not only the Ministry of Health took action towards mitigating AMR, but the Africa Centers for Disease Control and Prevention (Africa CDC) also launched its strategies in enhancing surveillance, preventing occurrence, restricting transmission and alleviating the risks of AMR in Africa in 2018. The CDC, in partnership with the Center for Disease Dynamics, Economics & Policy, helps healthcare workers regarding the correct dosage, usage, selection and duration of antimicrobial treatments recommended by the experts in treating bacterial infections and promoting the proper use of antimicrobials to mitigate the spread and occurrence of AMR [35]. Recently, the Food and Agriculture Organization (FAO) [36], World Organization for Animal Health (WHAH), WHO and other interest groups established the AMR Multi-Partner Trust Fund to help low-income countries combat the menace of AMR. The fund will operate for five years, until 2024, and has received a $5 million initial donation from the Dutch government. The immediate financing demand is $70 million, which will provide nations with technical assistance in developing NAPs and scaling up local action [37].

No doubt, African governments and partner agencies’ undertaking have been a great collaborative effort for controlling AMR in African countries. Despite the initiatives and collaborative efforts of the governments, there still lies the possibility of buying antibiotics with no prescription. Uncontrolled intake of antibiotics may further escalate the emergence of AMR. Thus, more actions must be taken to address these underlying concerns.

## AMR surveillance systems in Africa

Tackling AMR requires effective surveillance systems to monitor the trend of resistance and generate data about the extent of the burden, without which it would be impossible to guide policies and strategies to limit further resistance development and check our progress toward curbing the spread of resistance [38]. Therefore, clinical judgments regarding empirical therapy should be based on knowledge about the likely pathogens and their antibiotic sensitivity [39]. This information is gathered over time, partly through clinical practice but more objectively and solidly through surveillance [39]. Surveillance has been defined as "the continual systematic collection, analysis, and interpretation of health data essential to the planning, implementation, and evaluation of public health practice, closely integrated with timely dissemination of these data to informed decision making [39]." The core components of national AMR surveillance systems have been described by WHO [7]. Due to a paucity of continent-wide surveillance data, understanding the true incidence and effect of AMR in Africa is difficult, particularly for infections requiring specialized testing procedures. Whereas industrialized countries use a combination of genomic and laboratory monitoring methods, the bulk of African countries rely only on laboratory-based surveillance. While most African nations acted upon the WHO global strategy to develop a surveillance action plan for AMR, the openness about AMRs NAPs progress and budget allocations is extremely limited [10, 29].

**Fig. 1:**
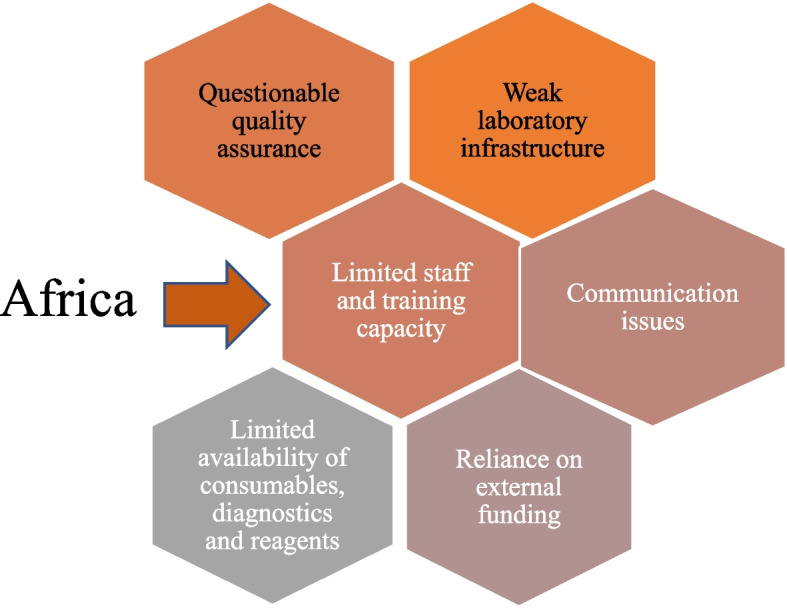
AMR surveillance challenges in Africa

Implementing a NAP for AMR surveillance from a One Health approach is pivotal to extending the useful life of antibiotics in African countries, improving access to diagnostic laboratories, monitoring and control of resistance, as well as better regulation and education of the general public, clinicians and prescribers, and veterinarians. However, as with other continents, the implementation of effective and sustained AMR surveillance programs in Africa incurred many challenges. Among these challenges are a lack of funding for microbiological laboratory operations, weak supply chains of components, and a lack of personnel properly trained in AMR surveillance. Facility management is also not sufficiently committed to embracing AMR as a healthcare issue [37]. And even where facilities exist, underestimation is common. There is also the problem of substandard diagnostic equipment and laboratory consumables that circulate throughout Africa, leading to false-negative results and unwarranted antibiotic treatments [40]. Figure 1 summarizes the challenges associated with AMR surveillance in Africa.

## Challenges hindering the implementation of Global Antimicrobial Resistance Surveillance System (GLASS) in Africa

As a response to the need to step up the fight against AMR, the WHO developed the GLASS in 2015 and encouraged member states to enroll. In January 2017, The Africa CDC was established to assist African governments in beginning surveillance for significant infectious disease risks, particularly resistance [40, 41]. While a few African countries currently conduct effective routine AMR surveillance, others do not have surveillance systems in place, according to the information available in a recent WHO GLASS report [7]. As of March 2020, 21 of the 47 African countries had completed enrolment, and 49 laboratories from 28 countries were participating in the WHO antimicrobial susceptibility testing (AST) program. The susceptibility testing was guided by the WHO list of pathogens of concern to assist in the monitoring of resistance and to guide research and development of novel antibiotics. These bacteria have the ability to develop novel strategies to resist treatment and can transmit genetic information that helps other bacteria to become drug-resistant as well [7, 42]. The GLASS-AMR seeks to support national capacity development to monitor the status of national surveillance systems for AMR. It is a standardized approach to ensuring the collection, analysis, and sharing of AMR data among countries [7]. The Africa CDC established the Anti-Microbial Resistance Surveillance Network (AMRSNET) to guide the implementation of GLASS from a one-health perspective. However, this has recorded little success as priority is not given to food-producing animals, which are considered key reservoirs of resistant pathogens [40].

Africa faces a key implementation challenge due to the lack of breakthrough technology that would enable data transfer to the WHO GLASS database; even where such technologies exist, transmissions are intermittent [10]. Ethiopia, for example, has a NAP for laboratory-based antibiotic resistance surveillance. The surveillance system comprises a network that connects the national reference laboratory to surveillance sentinel stations. After the implementation began, the microbiology lab experienced challenges with the electronic capture and transmission of data, the supply chain for the lab, and communications [43]. In Uganda, although laboratory-based surveillance exists, but it is not consistently deployed across the country. In addition, analog methods were used for analysis, reporting, and transmission [44]. This points to the necessity of a concerted effort to ensure the availability of resources for regular lab-based surveillance of AMR. However, South Africa has made a decent start in terms of AMR surveillance with both public and private surveillance systems in place, but it can and should be enhanced [38]. Surveillance data for most AMR are laboratory-based and hence organism-centric, making it difficult to distinguish between colonization and infection with AMR organisms. The significant problem is that AMR surveillance is currently restricted to a small number of hospital settings, which does not reflect the scope of AMR in South Africa [38]. In Zimbabwe, the deployment of AMR monitoring initiatives is hampered by a lack of contacts and AMR surveillance training among healthcare workers. Moreover, this is in addition to the country’s focus on HIV and TB while neglecting microbiology laboratories and other AMR programs. Kenya and Tanzania have similar challenges due to insufficient finance, a lack of qualified personnel, multisectoral coordination, and communication to enable real-time data exchange [45]. In Cameroon, diagnostic test kits are very scarce or non-existent, and the country lacks technical resources in the field of microbiology [45, 46]. As a result, clinical isolates cannot be tested for susceptibility. As a step toward addressing the technical gap, the United States CDC, in collaboration with the Africa Society for Laboratory Medicine (ASLM) deployed a team of experts to help with training the staff and implementing an AMR checklist [45]. However, despite these efforts, challenges associated with the implementation of effective and sustainable AMR surveillance in Africa, such as inadequate resources and poor supply chains for chemicals and reagents for laboratory assays, inadequate and poorly trained staffers as well as a lack of commitment from the side of hospital management, still remain [37].

## AMR diagnostic tools available in Africa

As efficient as surveillance and stewardship programs are in fighting the AMR crisis, their success hinges largely on the availability of rapid, reliable and indeed affordable antimicrobial susceptibility testing methods and technologies..

The AMR detection methods can be broadly classified into phenotypic such as disk diffusion, agar dilution, gradient test, and broth microdilution, VITEK 2 COMPACT andSensititre™ on the one hand, and molecular methods such as polymerase chain reaction (PCR)-based assays, isothermal amplification, methods, DNA microarrays and Whole Genome Sequencing (WGS), on the other. These methods have been widely reviewed [47–50]. In this section, therefore, we will focus on the availability and deplorability in the context of Africa since diagnostic tests are regarded as crucial tools in the success of the fight against AMR. Indeed, both the absence of rapid and inaccurate detection of pathogens constitute a major problem, forcing clinicians to rely on empirical antibiotic therapies, usually using antibiotics with a broad spectrum. This situation further fuels the spread of AMR, which, in turn, results in higher mortality rates, prolonged hospitalization and increased associated costs [49, 50]. Moreover, poor detection tools mean resistant pathogens continue to spread from humans and animals to the environment.

## Phenotypic AMR detection methods

Phenotypic methods, to begin with, have received wide acceptance in many African countries, both in research labs and in regular diagnosis. Their wide deployment in African countries has been linked to their simplicity, cost-effectiveness and non-laborious nature [51, 52]. These tests include the double disc synergy test (DDST), combined disc method (CDM), Epsilometric test (E-test), disc replacement method (DRM), Broth Microdilution (BMD), and isoelectric focus (IEF). Several studies in Ethiopia [53], South Africa [54], Nigeria [55], Egypt [56] and Ghana [57], among others, indicated the availability of phenotypic methods for AMR detection in Africa. Similarly, in a systematic review conducted in part of northern Nigeria, a west African state, the use of DDST was found to be 90.2%, only 1.6% reported the use of CDM and E-test, but 2% combined both DDST and CDM for testing ESBL [58]. In addition, Bashir et al. [59] used the Kirby-Bauer disc diffusion method in three tertiary hospitals in Kano state to investigate AMR, northwestern Nigeria. Despite the inefficiency of the Kirby-Bauer disc diffusion technique, the authors reported 100% resistance to cotrimoxazole, pefloxacin, amoxicillin, and imipenem by *Acinetobacter* spp and more than 80% by nosocomial pathogens.

Disc diffusion assay is a qualitative method in which antimicrobial substances diffuse over the media to identify the microorganisms' resistance profile. However, it’s time-consuming and some antimicrobial substances do not diffuse well in the media. Minimum Inhibitory Concentration (MIC), on the contrary, is a quantitative technique that determines the antimicrobial susceptibility profile of particular microorganisms but cannot detect AMR in non-culturable microbes, though it’s simple and inexpensive [56, 60]. BMD also is simple, affordable, accurate but time-consuming (16–24 h) like most other phenotypic methods [49]. Although the disc diffusion method has certain limitations, it is the most widely available phenotypic tool in most African laboratories [49, 55, 57, 61, 62]. In contrast, E-test combines both qualitative (disc diffusion method) and quantitative (MIC) methods containing drugs concentration gradient and calibration scale on either side. Its major setback, however, is time consumption but can detect AMR in fastidious organisms like *Haemophilus influenza* and *Mycobacterium bovis*, unlike disc diffusion method [60, 63, 64]. E-test has been often used to monitor the resistance of penicillin, tetracycline, and ciprofloxacin in *Neisseria gonorrhoeae* across the South African region and even other African countries [60, 65].

Similarly, meropenem + EDTA is employed for differentiating metallo-β-lactamase from serine carbapenemase. In contrast, meropenem /meropenem + phenylboronic acid) is widely used to classify *Klebsiella pneumoniae* carbapenemase (KPC) from other gram-negative bacteria (serine carbapenemase-producing) in many African countries [53, 66]. Imipenem-EDTA combined disk (MβL-CD) methods and modified carbapenem inactivation (mCIM) are used to phenotypically screen carbapenemases and MβLs production potentials [67]. Most of these methods are not adequately available in some African laboratories [52, 53, 68]. In addition, Carba NP test method detects carbapenemase in gram-negative bacteria. Conversely, the preparation and storage of reagents for a longer period are some of its limitations [53, 69]. This test has great specificity (100%) and sensitivity (100%) for class A (KPC, NMC-A, SME), class B ( metallo-β-lactamasesNDM, GIM, SPM, IMP, VIM), class D (OXA-48, OXA-181) carbapenemase producers [70]. Nevertheless, Carba NP method is not routinely available in most African laboratories [71, 72], even though the first emergence of carbapenemase-producing Enterobacteriaceae (CPE) in Sokoto state (Nigeria) and NDM-producing *Citrobacter freundii* in Nigeria were detected using the modified Carba NP method [73]. In addition, the Modified Hodge test is a simple and cost-effective phenotypic test that detects CPE [74–76] but is less specific and takes a longer time [77], and few reports indicated its usage in some African laboratories [78, 79]

Other phenotypic methods include immunochromatography and colorimetric assays. The colorimetric test is a method which detects AMR through pH and color changes as a consequence of bacterial hydrolyzing enzymes [49, 53]. These methods are less employed in African laboratories [47, 80]. In Africa, the most rapid, cost-effective and reliable tools for detecting AMR resistance genes are inadequate and this causes delays in the diagnostic procedures as well as prolongs the duration of the treatment [3, 53]. These shortcomings underscore the urgency of providing not only high-throughput, robust, sensitive, less-expensive, and efficient diagnostic tools but also very rapid [3, 60, 81].

## Molecular methods of AMR detection

Molecular methods are generally used to detect resistance genes or point mutations that cannot be identified using phenotypic methods, thereby ensuring accurate detection and supporting timely treatment [81]. These techniques are gradually set to replace the traditional phenotypic methods for their greater precision and rapidity [60, 81]. The most common molecular methods for the detection of AMR include PCR, DNA microarray including Verigene and FilmArray systems, WGS, Xpert MTB/RIF, Genotype MTBDRplus, MTBDRs, metagenomics and mass spectrometry using matrix-assisted laser desorption ionization-time of flight, [48, 81, 82]. Although most of these methods are not available in a resource-limited continent like Africa due to financial constraints, required expertise and political will, they are gradually gaining popularity in the last few years, especially in South Africa, Nigeria and Egypt [55, 83–85].

Out of all molecular methods mentioned, PCR is the most commonly used in Africa [54, 86–88]. Different types of PCR exist, such as conventional PCR, Multiplex PCR, and RT-PCR. Conventional PCR detects the presence of resistant genes such as *vanA*, *mecA*, *ampC* in enterococci and staphylococci but it is less sensitive for detecting point mutation within the gene, while the RT-PCR can do that, is the constraint to detecting oligonucleotide (short fragments of DNA) [81, 89]. Mahmoud et al. [90] employed multiplex PCR to detect the presence of carbapenemase genes (bla_KPC_, bla_IMP_, bla_NDM_, bla_SPM_, bla_VIM_, and bla_OXA-48_) in *Escherichia coli* isolated from a source of water supply in Khartoum, Sudan. The authors revealed that bla_OXA-48_ (15.5%) gene was commonly found followed by bla_SPM_ (8.8%) and bla_KPC_ (4.4%) genes, respectively. Among the different types of PCR, conventional PCR, is the most widely used in African research laboratories, though RT-PCR and multiplex PCR are also available to a lesser degree [91–93]. Despite many reports indicating the availability of PCR in Africa, it’s not adequately available for routine laboratory investigations [3, 94].

The DNA microarrays that detect bacterial diversity have now been used to detect AMR. In this method, specific nucleic acid molecules are identified with the aid of a short DNA sequence (oligonucleotide) attached to a solid surface [49]. However, the use of solid surfaces such as glass slides and fluorescent dyes makes it time-consuming and more expensive [81]. In order to demonstrate the availability of this technology in Africa, Fashae et al. [95] employed the microarray technique to detect the presence of bla_CTX-M15_ and bla_CTX-M9_ in *E coli* in Ibadan, Nigeria. Another application of this technique was reported for detecting carbapenemase, ESBL, and AmpC genes in three major tertiary care hospitals in Egypt [84]. Despite this, however, microarray tech is still not routinely available in many African laboratories.

Other DNA microarrays include Alere microarrays, Identibac microarrays as well as Verigene and FilmArray systems [48, 81]. Verigene and FilmArray systems are rapid molecular tests for detecting AMR genes in gram-negative bacteria. The FilmArray system is a very sensitive and rapid method but can only identify a single KPC gene, limiting it usage. Although these methods (Verigene and FilmArray systems) may sometimes not detect cephalosporin/carbapenem resistance genes, their rapid, sensitive and specific nature gives them priority over traditional phenotypic and molecular techniques [48]. The availability of these tools is typically reported in the USA and few Africa [48, 80].

Most recently, MTBDRs as a rapid molecular line probe test was used to detect resistance genes in extensively drug-resistant Tuberculosis in Cape Town, South Africa. The method is not only rapid but also sensitive and has overcome some challenges of using conventional culture-based AMR assay in resource-limited areas like Africa [96]. However, its major setback is the failure to detect any mutation that was not specifically designed for the assay [97]. Genotype MTBDRsl and MTBDRplus assays are alternative techniques to phenotypic methods. They detect resistance to second-line anti-tuberculosis drugs but are less specific and sensitive to detecting kanamycin, capreomycin and ethambutol resistance [97]. In the last few years, the use of Genotype MTBDRsl/MTBDRplus is getting widespread across African laboratories as part of the global effort for combating tuberculosis TB and monitoring multidrug-resistant TB [98]. The specificity of MTBDRsl was reported as 85 percent [99]. These methods (MTBDRsl/MTBDRplus) were developed to facilitate the easy identification of tubercle bacilli and to replace the old-line probe assay technique [100].

Although most advanced molecular methods are reported in developed countries, some studies have reported the use of WGS, matrix-assisted laser desorption ionization-time of flight mass spectrometry (MALDI- TOF MS) and in silico multilocus sequence types (MLST) for AMR detection in Africa [51, 88, 101]. WGS detects mutations and genes responsible for AMR, just like PCR and Microarray, and has the potential to cover specific target genes and variants with high sensitivity [81]. Ingle et al. (2018) determined fluoroquinolones and nitrofurantoin resistance genes in *E. coli* using WGS across south Asia and sub-Saharan Africa. Over forty acquired AMR genes have been identified, including point mutations (two in gyrA, one in parC (both gyrA and parC are resistant to quinolone), nfsA (resistance to nitrofurantoin) and transposons [102]. The use of WGS for the detection of AMR in Africa despite financial constraints is gradually increasing [65, 103–105]

Similarly, MALDI- TOF MS was initially used to identify microbial species but due to its specificity and cost-effectiveness, it’s now employed for AMR screening in food-borne pathogens using specific biomarkers [106, 107]. MALDI- TOF MS has been used to detect AMR in Enterobacterales, non-fermenting gram-negative and gram-positive bacteria, mycobacteria and anaerobic bacteria [108]. As best as we can tell, no study has reported the use of this technique for routine laboratory investigation for AMR in Africa. Despite its robustness, MALDI-TOF MS poorly screened *Campylobacter jejuni* for ampicillin, kanamycin, gentamycin, erythromycin, and streptomycin resistance due to its unbalanced data set, which may not be applicable in clinical laboratories [106]. MLST, on the other hand, detects resistance genes using MLSTFinder and MLST allele sequence as well as PubMLST.org data profile specifies the sequence types (STs). The in silico detection is conducted using ResFinder with 90% and 60% identity thresholds for maximum and minimum length, respectively. The filter retains genes possessing maximum sequence identity and coverage, whereas overlapped genes are screened out. The first gene (blaCTX-M-55) among ESBL reported in animals and humans in Nigeria was detected using WGS and MLST [109]. However, MLST technology is still in the infancy stage in African laboratories [101, 109–111]. Recently, there has been the emergence of automated and semi-automated techniques that integrated microdilution susceptibility testing for rapid identification of bacteria and AMR/AST. These include VITEK 2 Systems, Phoenix System, MicroScan WalkAway plus System, MicroScan AutoScan 4 and MicroScan WalkAway System [49, 57]. Nevertheless, each of these methods has merit and demerit and the output may differ according to the software, antimicrobial agent and card used [49]. The facilities for these techniques are very scarce in Africa [54, 112]. Integrating these automated techniques with molecular methods may go a long way in improving rapid AMR detection.

## Conclusion

We have uncovered some level of progress in the areas of AMR stewardship, surveillance programs and deployment of various kinds of phenotypic and molecular diagnostic tools across Africa, with South Africa the lead in all aspects. However, in most parts of Africa, particularly in remote and primary healthcare settings, not much has been done. The major challenges of lack of proper microbiological diagnostic tools for the identification of AMR, particularly in routine diagnostic labs continue to linger and may continue to jeopardize national and international AMR containment efforts. As such, AMR continues to remain a great challenge, and as a consequence, solid stewardship and surveillance programs, as well as easy-to-use, reliable, and cost-effective diagnostic tools, are in demand on the continent. The lack of affordable AMR diagnostic tools, stewardship and surveillance programs implies that resistant pathogens will continue to go undetected and may continue to spread from the hospital environment to the community. Often, these kinds of antibiotic-resistant pathogens are detected when treatment failure becomes eminent. Thus, more needs to be done in the areas of funding, legislation and enforcement on the side of government, more advocacy on the side of CSOs and indeed continuous AMR surveillance and adherence to the international guidelines at the institutional levels. These measures will significantly reduce the emergence, transmission, and potential dangers of AMR both within facilities and within communities.

## Data Availability

Not applicable.
